# AFRbase: a database of protein mutations responsible for antifungal resistance

**DOI:** 10.1093/bioinformatics/btad677

**Published:** 2023-11-08

**Authors:** Aakriti Jain, Neelja Singhal, Manish Kumar

**Affiliations:** Department of Biophysics, University of Delhi South Campus, New Delhi 110021, India; Department of Biophysics, University of Delhi South Campus, New Delhi 110021, India; Department of Biophysics, University of Delhi South Campus, New Delhi 110021, India

## Abstract

**Motivation:**

Fungal pathogens are known to cause life threatening invasive infections with rising global mortality rates. Besides, the indiscriminate use of antifungals in both clinics and agriculture has promoted antifungal drug resistance in the last decade. Fungi can show drug resistance by a variety of mechanisms. But primary driver in all these hitherto documented mechanisms is stable and heritable point mutations in the key proteins. Therefore, cataloguing mutations that can confer resistance is the first step toward understanding the mechanisms leading to the emergence of antifungal resistance.

**Results:**

In the present, work we have described a database of all the mutations responsible for antifungal resistance. Named as antifungal resistance database (AFRbase), it is better than the existing databases of antifungal resistance namely, FunResDB and MARDy which have a limited scope and inadequate information. Data of AFRbase was collected using both text mining and manual curation. AFRbase provides a separate window for visualization of mutations in the 2D and 3D formats making it easy for researchers to analyze the mutation data and ensures interoperability with other standard molecular biology databases like NCBI and UniProtKB. We hope AFRbase can be useful to both clinicians and basic biomedical scientists as we envision it as an important resource for genotypic susceptibility testing of fungi and to study/predict the course of evolution of antifungal resistance. The current version of AFRbase contains manually curated 3691 unique mutations present in 29 proteins of 32 fungal species along with the information of drugs against which resistance is caused.

**Availability and implementation:**

AFRbase is an open access database available at http://proteininformatics.org/mkumar/afrbase/.

## 1 Introduction

Besides being ubiquitous in the environment, fungi are important components of the microflora of the human skin, lungs, genitourinary, oral, and gastrointestinal tracts (Rolling *et al.* 2020). In immunocompromised patients, commensal fungi might compound to invasive fungal infections by systematic translocation (Zhai *et al.* 2020). Prior to COVID-19 pandemic, fungal infections were associated with approximately 1.5–1.7 million annual deaths and 300 million superficial infections (Brown *et al.* 2012, Denning and Jugessur 2016, Bongomin *et al.* 2017, Kainz *et al.* 2020, Rodrigues and Nosanchuk 2020). During COVID-19 pandemic, secondary fungal infections with black and yellow fungi were a major factor underlying complicated clinical cases. Besides human infections, fungi are also responsible for major losses in agricultural activities and global food production due to severe crop damage. Global estimates indicate that fungi are the highest threat for animal–host and plant–host species, accounting for a majority of pathogen-driven host losses (Almeida *et al.* 2019).

The widespread and indiscriminate use of antifungals both in clinical and in agricultural setups has resulted in rapid increase of drug-resistant fungi. Antifungal drug resistance is a matter of grave concern due to limited choice of drug targets as fungi are eukaryotes, like humans. Earlier, antifungal resistance was neglected as a threat to public health (Fisher *et al.* 2022). However, inclusion of fungi as a priority pathogen in the 2019 report of Center for Disease Control and Prevention (CDC https://www.cdc.gov/drugresistance/biggest-threats.html^2022^) and the launch of Global Antimicrobial Resistance and Use Surveillance System (GLASS) by the World Health Organization [Global Antimicrobial Resistance and Use Surveillance System (GLASS) Report 2021] has highlighted the fungal disease burden and paved the way for streamlining the process of fungal data collection, analysis, and sharing. Various reports have also highlighted the need of a fungal repository for better surveillance of infectious fungal species (Chowdhary and Sharma 2020, White 2021, Gangneux *et al.* 2022). The recent release of the fungal priority pathogen list by WHO has further accelerated research, development and public health action in this direction (World Health Organization 2022).

The inherent biological complexity of pathogenic fungi, challenges in their culture and continuous increase in fatalities have created a gap between clinical and research activities. There is scarcity of antifungal drugs and only three major classes of antifungals—polyenes, azoles, and echinocandins are available. The efficacy of these antifungals has been compromised by the increasing antifungal resistance. The availability of limited treatment options and shortcomings of the existing ones is further worsened by lack of published fungal data, except for drug-resistant *Candida* and *Aspergillus* species. Fungi may show drug resistance by alteration or overexpression of primary drug targets, up-regulation of multidrug transporters and cellular stress response pathways, and efflux of drug from the fungal cells. Nevertheless, the fundamental factor that contributes to antifungal resistance in all these mechanisms is the presence of stable and heritable point mutations in key proteins. Antifungal resistance threatens the limited antifungal armamentarium and affects clinical outcomes by delaying mycological clearance, increasing breakthrough infections, relapse and excess mortality. Despite the clinical and economic relevance of antifungal drug resistance, this subject remains poorly studied, at least in comparison to the similar issue of bacterial drug resistance. Till date, only two databases of antifungal resistance are available. These databases are FunResDB (Weber *et al.* 2018) and MARDy (Nash *et al.* 2018). However, the depth and information coverage of both these databases is limited. FunResDB provides information on 79 variants of only *Cyp51A* gene of *Aspergillus fumigatus*. MARDy, though contains more information than FunResDB, provides information on 232 mutations in 18 fungal genes across fungal genera/species. Thus, both these databases have a limited scope and inadequate information on antifungal resistance. To address the lacunae in the existing databases and to provide a comprehensive and updated information on all the mutations responsible for antifungal resistance (reported till date, as on 30 June 2023) we have created a database, antifungal resistance database (AFRbase). The “One Health” approach was applied while collating the data for AFRbase, implying the database covers all the fungal pathogens infecting plants, animals and humans along with the mutations in their drug targets. Moreover, integration of data analysis modules using in-house developed R applications to visualize mutation hotspots leading to drug resistance, is an add-on feature of AFRbase.

## 2 Materials and methods

### 2.1 Database content and implementation

The current version of AFRbase provides information of a total of 3691 mutations which lead to resistance against 37 antifungal drugs. Each data entry is linked to the research paper in which that particular mutation and resistance for the antifungal drug was reported. The data was initially collected through manual text-based search. Using the MeSH (Medical Subject Headings) terms of the research papers collected by manual search, a text mining search pipeline was developed to include all the reported antifungal resistance mutations. Each entry in the database has been enriched with information from public databases like NCBI, UniProtKB, and PDB. Further, the disease information related to fungi was also integrated using the information from fungal repertoires such as, Fungal Genetics Stock Center (McCluskey *et al.* 2010), Fungicide Resistance Action Committee (Leadbeater 2012), and Global Action Fund for Fungal Infection (GAFFI, https://gaffi.org). Also, to facilitate the search of proteins/mutations in which a user might be interested in further research, we have incorporated the BLAST tool. The inner framework of AFRbase has been built using MySQL (http://www.mysql.org), Perl (http://www.perl.org), and Apache (http://www.apache.org). The interface (Supplementary Data) was designed in HTML using CSS and Javascripts in a CentOS Linux environment along with a local installation of BLASTp for sequence analysis. For data visualization R packages, Javascript modules, and JMol (https://jmol.sourceforge.net/) were used.

### 2.2 Visualization and analysis tools

For identification and analysis of propensity of various mutations in an antifungal protein, two visualization, and analysis tools have been incorporated in the AFRbase. These are:

#### 2.2.1 Mutation plots

**Figure 1.**
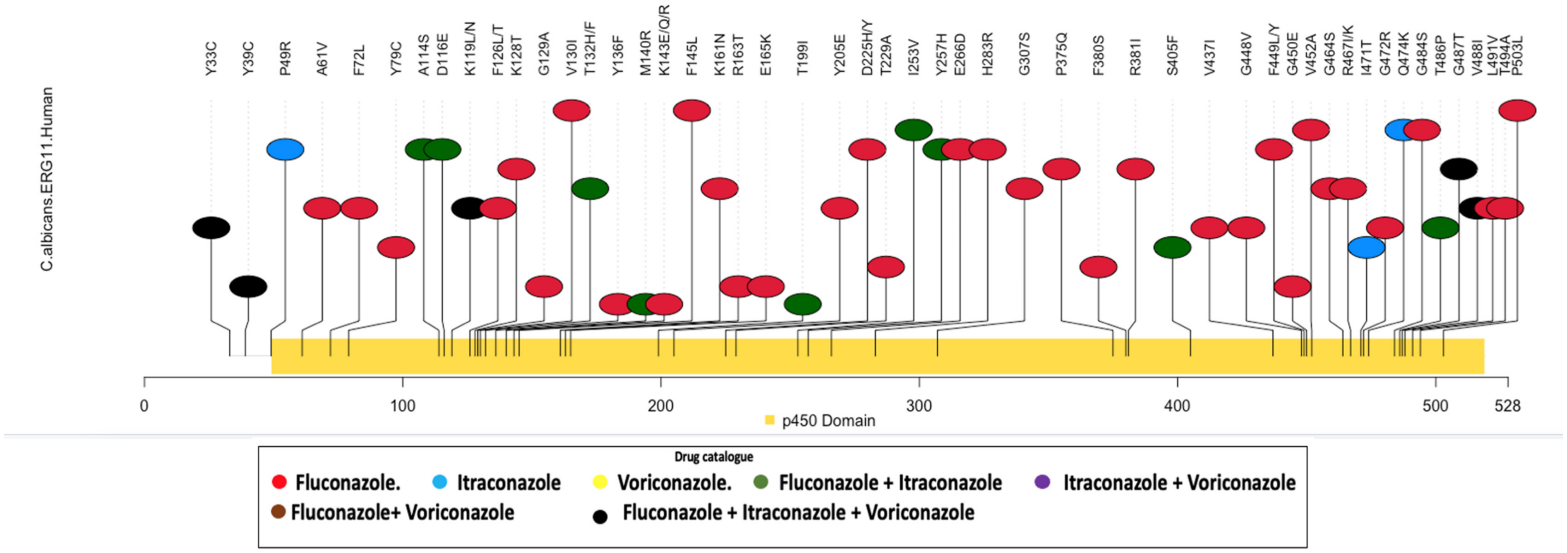
MutHotPlot of the fungal protein “ERG11” constructed using an in-house developed R application (MUTe_DRUG_Spot). The *X*-axis represents the amino acid positions in the ERG11 protein. Yellow bar shows Pfam domain information. *Y*-axis represents mutation instances through the lollipop-like structures at their specific loci. The color coding of the mutation instances corresponds to the drug against which resistance is conferred by that particular mutation. For example, the mutation Y33C in ERG11 results in resistance against Fluconazole, Voriconazole, and Itraconazole (reprsented in black).

To visualize all the reported mutations of a protein and study their impact on antifungal resistance we have created lollipop plots using an in-house developed R application. In these plots lollipop-like structures represent mutations at specific loci across the length of the protein and the color of the lollipop represents the specific drugs against which resistance is conferred by the respective mutations. The plot is called as MutHotPlot. An example of MutHotPlot for fungal gene “ERG11” is shown in Fig. 1.

#### 2.2.2 JMol-based molecular visualization

**Figure 2.**
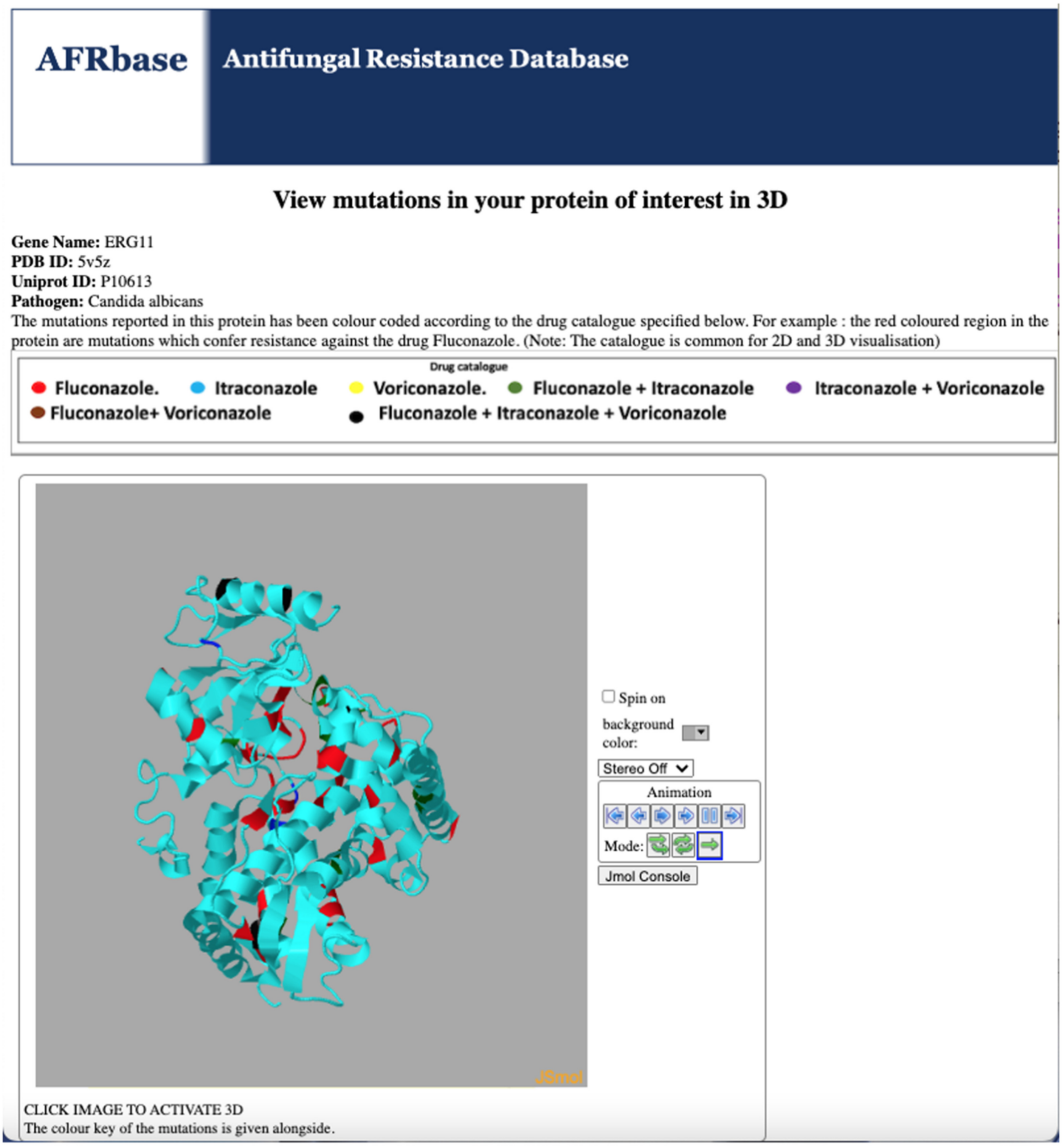
Snapshot of the 3D visualization of protein mutations in a fungal protein (in this case, ERG11) embedded as a JSmol object in AFRbase.

To visualize the location of mutation(s) in each protein in the 3D structure, we have plugged in the JSmol viewer (http://www.jmol.org/) in AFRbase (Fig. 2).

As mutation(s) were numbered on the basis of protein sequences, there might be differences in numbering of amino acid(s) in protein sequences in UniProt and their corresponding PDB records. Therefore, to bring uniformity in the position of mutation(s) at the protein sequence and structure, we used PDBrenum (Faezov and Dunbrack 2021), a web server that corrects sequence numbering to facilitate interoperability between standard databases. We fed UniProt sequences and obtained the mutations on numbered PDB records using this server. This information was fed in the JMol script to color the mutated regions as per our drug catalog. These edited structures were obtained as JMol objects and linked to our database. Through this the entire set of identified mutations on a protein can be visualized in 3D.

## 3 Discussion

Currently, only two antifungal resistance databases are available, FunResDB and MARDy. FunResDB is a webresource which provides information only for 79 variants of a single gene—*CYP51A* which causes azole resistance in *Aspergillus fumigatus.* MARDy is more informative than FunResDB as it provides information about 232 mutations in 18 genes across 27 fungal genera/species resulting in resistance for 29 antifungal drugs. Compared to both these, AFRbase is a more advanced and updated antifungal resistance database as it covers all the mutations associated with antifungal resistance, reported till date (30 June 2023). As of now, AFRbase provides information on 3691 mutations in 29 genes across 32 fungal genera/species resulting in resistance for 37 antifungal drugs. Besides being old, MARDy was created using manual keyword-based literature search while AFRbase was created using both text mining and manual curation. Hence, the latter has more information on mutations information about mutations from MARDy as it lists all the information that cause antifungal resistance. Moreover, it is difficult to comprehend in downloadable tabular formats with limited search options. On the other hand, besides providing user-friendly search options AFRbase provides a separate window for visualization of mutations in the 2D and 3D formats making it easy for researchers to analyze the mutation data. AFRbase also provides information about identifiers obtained from other standard molecular biology databases like NCBI and UniProtKB promoting interoperability between AFRbase and other databases which is absent in MARDy.

Mutations are the most important factor underlying evolution and natural selection of living organisms as they capacitate them to survive in adverse conditions. In fungi, all the variations/mutations in drug target genes might not lead to antifungal resistance. Hence, a repository of mutations which confer antifungal resistance, like AFRbase will be an important resource for genotypic susceptibility testing of fungi and study/predict the evolution of antifungal resistance. We hope AFRbase will be useful for both clinicians in deciding and basic biomedical scientists researching on antifungals and antifungal resistance.

### 3.1 Future updates

We plan to release future updates of our database which shall comprise a BLAST tool for mutation detection and a BLAST tool for primer evaluation. In addition, we plan to translate our mutation hotspot forming code into an online tool and release it in the public domain. We believe the mutation tool will provide researchers a flexible gateway to plot, visualize and analyze their mutation data.

## Supplementary Material

btad677_Supplementary_Data
