# Supplementary material for: AFRbase: a database of protein mutations responsible for antifungal resistance
Source: Bioinformatics. 2023 Nov 8;39(11):btad677. doi: 10.1093/bioinformatics/btad677 (PMC10656092; doi:10.1093/bioinformatics/btad677)
Supplement: btad677_Supplementary_Data [file btad677_supplementary_data.pdf]

AFRbase

Antifungal Resistance Database

Home Search Blast 2D View 3D View Submit Statistics Contact Help

Browsing Categories

Browse by Gene

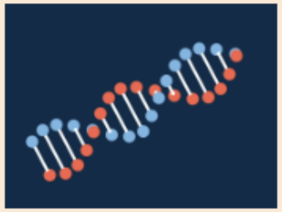

Browse by Drug

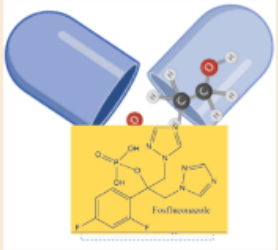

Browse by Pathogen

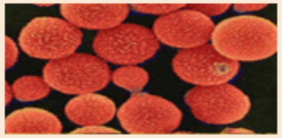

OBJECTIVES OF AFRbase

AFRbase-Antifungal Resistance Database aims to serve as a curated web resource for efficient manipulation, browsing and analysis of resistance conferring mutations in proteins of high risk fungal pathogens. It provides a convenient, user-friendly interface to query, browse and visualize the mutation patterns of key fungal proteins along with their parent genes and other allied information fetched from published literature. AFRbase has been made with the target of serving as a comprehensible resource with its utility for clinicians and researchers alike.

As of now, our database documents about 3691 amino-acid mutations affecting the resistance profiles of diverse fungal pathogens against standard antifungal drugs.

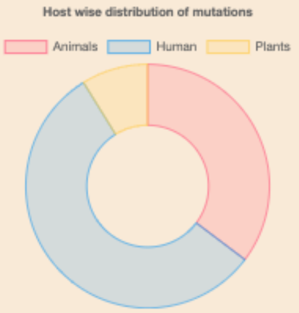

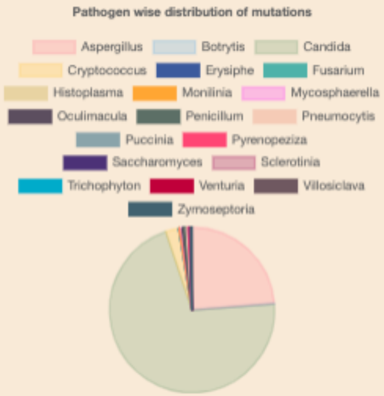

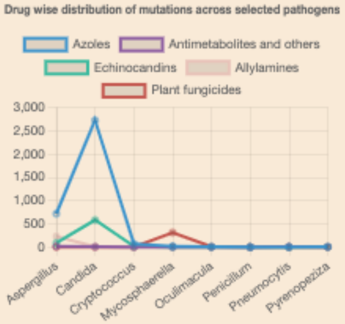

DATA VISUALISATION TOOL

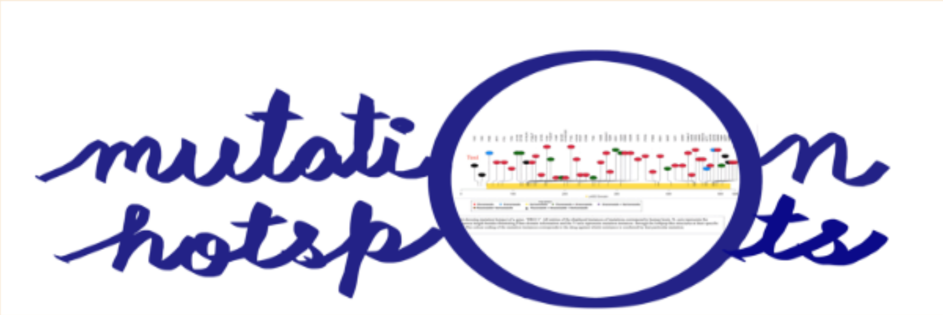

To plot your own list of mutations and drugs through our tool click "here".

AFRbase in Numbers

Host Organisms
3

Fungal species
32

Genes
29

Drugs
37

Diseases
31

Mutations
3691

Research Articles
>6787

Acknowledgements

International Mycological Association, Pfam, NCBI, FRAC, UniProt, Gaffi

To cite our database kindly see: Link

© Department of Biophysics University of Delhi South Campus.

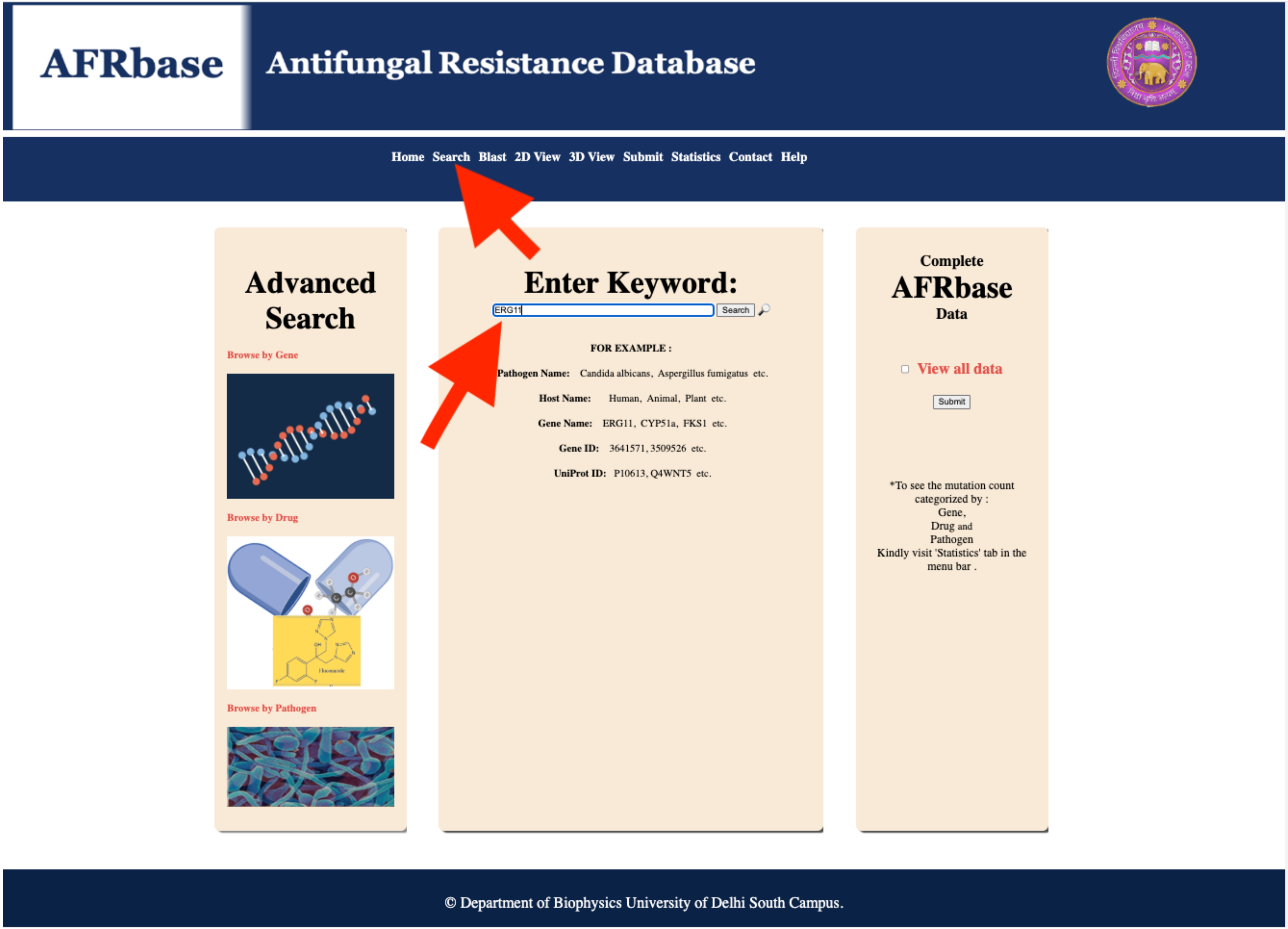
AFRbase
Antifungal Resistance Database
Home Search Blast 2D View 3D View Submit Statistics Contact Help
Advanced Search
Browse by Gene
Browse by Drug
Browse by Pathogen
Enter Keyword:
ERG11
Search
FOR EXAMPLE :
Pathogen Name: Candida albicans, Aspergillus fumigatus etc.
Host Name: Human, Animal, Plant etc.
Gene Name: ERG11, CYP51a, FKS1 etc.
Gene ID: 3641571, 3509526 etc.
UniProt ID: P10613, Q4WNT5 etc.
Complete AFRbase Data
View all data
Submit
*To see the mutation count categorized by : Gene, Drug and Pathogen Kindly visit 'Statistics' tab in the menu bar .
© Department of Biophysics University of Delhi South Campus.

AFRbase

Antifungal Resistance Database

• Home • Search • Blast • 2D View • 3D View • Submit • Statistics • Contact • Help

Search Results

Click **AFRbase ID** for more information

AFRbase ID	Fungal Pathogen	Host	Gene ID	Gene Name	Mutation Count per gene	Uniprot Entry	AA Mutations	Drug
6	Aspergillus flavus	NA	NA	ERG11	1357	NA	G54R	voriconazole
9	Aspergillus flavus	NA	NA	ERG11	1357	NA	G54R	itraconazole
12	Aspergillus flavus	NA	NA	ERG11	1357	NA	G54R	posaconazole
15	Aspergillus flavus	NA	NA	ERG11	1357	NA	G54R	azole
18	Aspergillus flavus	NA	NA	ERG11	1357	NA	G54R	isavuconazole
21	Aspergillus flavus	NA	NA	ERG11	1357	NA	G54R	fluconazole
36	Aspergillus flavus	NA	NA	ERG11	1357	NA	M220I	azole
39	Aspergillus flavus	NA	NA	ERG11	1357	NA	M220I	voriconazole
42	Aspergillus flavus	NA	NA	ERG11	1357	NA	M220I	terbinafine
45	Aspergillus flavus	NA	NA	ERG11	1357	NA	M220I	micafungin
48	Aspergillus flavus	NA	NA	ERG11	1357	NA	M220I	itraconazole
51	Aspergillus flavus	NA	NA	ERG11	1357	NA	M220I	posaconazole
54	Aspergillus flavus	NA	NA	ERG11	1357	NA	M220I	miltefosine
57	Aspergillus flavus	NA	NA	ERG11	1357	NA	M220I	isavuconazole
60	Aspergillus flavus	NA	NA	ERG11	1357	NA	M220I	anidulafungin
193	Aspergillus fumigatus	NA	NA	ERG11	1357	NA	G54R	voriconazole
196	Aspergillus fumigatus	NA	NA	ERG11	1357	NA	G54R	itraconazole
199	Aspergillus fumigatus	NA	NA	ERG11	1357	NA	G54R	posaconazole
202	Aspergillus fumigatus	NA	NA	ERG11	1357	NA	G54R	azole
205	Aspergillus fumigatus	NA	NA	ERG11	1357	NA	G54R	isavuconazole
208	Aspergillus fumigatus	NA	NA	ERG11	1357	NA	G54R	fluconazole
209	Aspergillus fumigatus	NA	NA	ERG11	1357	NA	Y132F	fluconazole
211	Aspergillus fumigatus	NA	NA	ERG11	1357	NA	Y132F	anidulafungin
213	Aspergillus fumigatus	NA	NA	ERG11	1357	NA	Y132F	caspofungin
215	Aspergillus fumigatus	NA	NA	ERG11	1357	NA	Y132F	voriconazole
217	Aspergillus fumigatus	NA	NA	ERG11	1357	NA	Y132F	flucytosine
219	Aspergillus fumigatus	NA	NA	ERG11	1357	NA	Y132F	micafungin
221	Aspergillus fumigatus	NA	NA	ERG11	1357	NA	Y132F	itraconazole
223	Aspergillus fumigatus	NA	NA	ERG11	1357	NA	Y132F	posaconazole
225	Aspergillus fumigatus	NA	NA	ERG11	1357	NA	Y132F	azole
239	Aspergillus fumigatus	NA	NA	ERG11	1357	NA	G54E	caspofungin
241	Aspergillus fumigatus	NA	NA	ERG11	1357	NA	G54E	voriconazole
243	Aspergillus fumigatus	NA	NA	ERG11	1357	NA	G54E	itraconazole
245	Aspergillus fumigatus	NA	NA	ERG11	1357	NA	G54E	posaconazole
247	Aspergillus fumigatus	NA	NA	ERG11	1357	NA	G54E	azole
249	Aspergillus fumigatus	NA	NA	ERG11	1357	NA	G54E	fluconazole
337	Aspergillus fumigatus	NA	NA	ERG11	1357	NA	M220I	azole
340	Aspergillus fumigatus	NA	NA	ERG11	1357	NA	M220I	voriconazole
343	Aspergillus fumigatus	NA	NA	ERG11	1357	NA	M220I	terbinafine
346	Aspergillus fumigatus	NA	NA	ERG11	1357	NA	M220I	micafungin
349	Aspergillus fumigatus	NA	NA	ERG11	1357	NA	M220I	itraconazole
352	Aspergillus fumigatus	NA	NA	ERG11	1357	NA	M220I	posaconazole
355	Aspergillus fumigatus	NA	NA	ERG11	1357	NA	M220I	miltefosine
358	Aspergillus fumigatus	NA	NA	ERG11	1357	NA	M220I	isavuconazole
361	Aspergillus fumigatus	NA	NA	ERG11	1357	NA	M220I	anidulafungin
424	Aspergillus fumigatus	NA	NA	ERG11	1357	NA	S663P	fluconazole

More Information

For 2D and 3D view of mutations, navigate to the '2D View' and '3D View' tabs in the menu bar

Features	
AFRbase ID	6
Fungal Pathogen	Aspergillus flavus
Host	NA
Disease caused	NA
Mode of transmission	NA
GenBank ID	NA
Gene ID	NA
Gene Name	ERG11
Gene Locus	NA
Uniprot Entry*	NA
Sequence	NA
Amino Acid Mutations	G54R
Drug against which resistance is gained	voriconazole
Functional Annotation	NA
PubMed ID*	34252311; 31315952; 31285229; 29891597; 29846581; 29684150; 29437612; 29263059; 28893791; 27664994; 25885568; 22581906; 20831364; 15563516
Reference	NA

Download Text File

Note: * are hyperlinked to UniProt and PubMed

© Department of Biophysics University of Delhi South Campus.

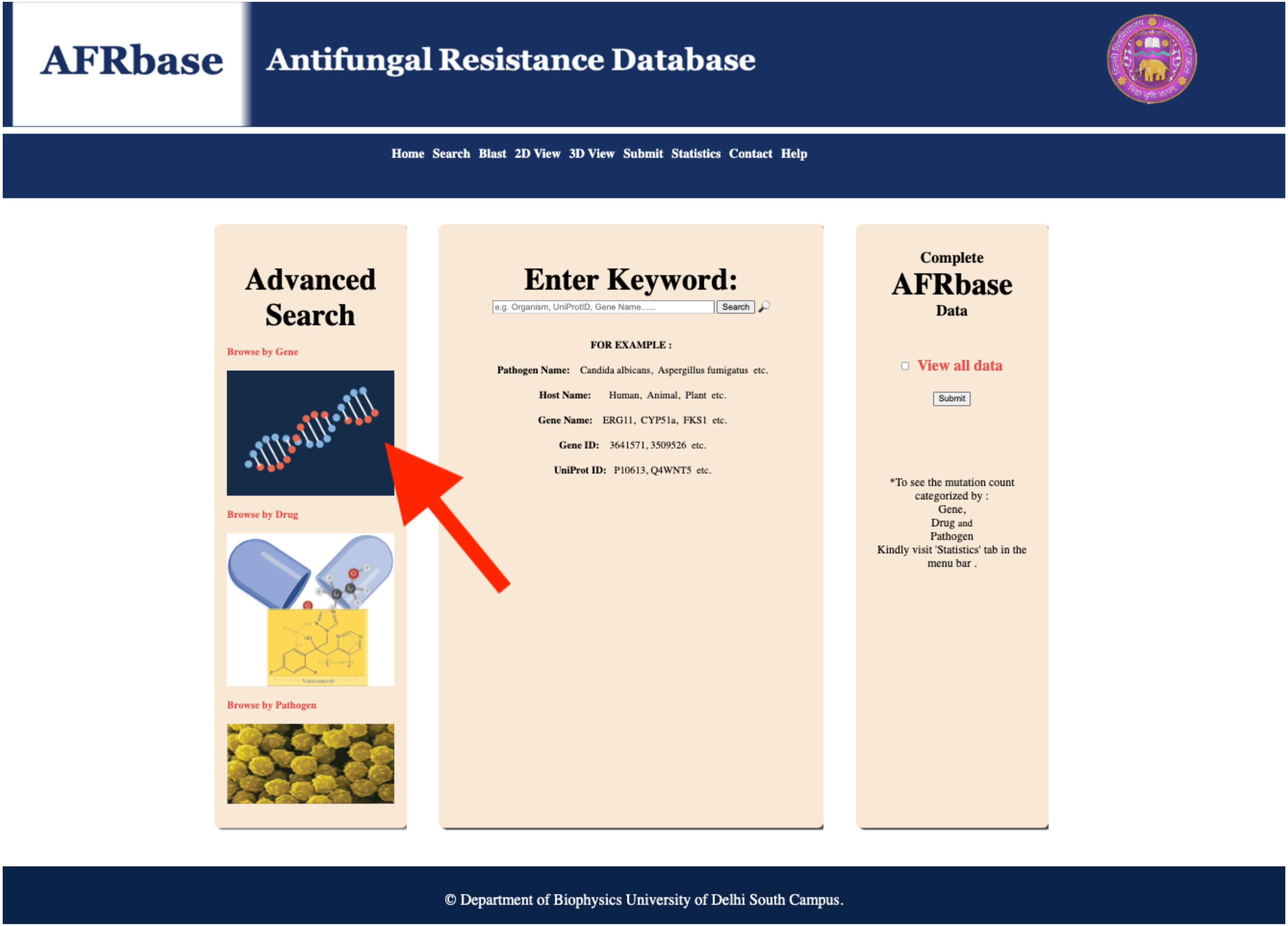
AFRbase
Antifungal Resistance Database
Home Search Blast 2D View 3D View Submit Statistics Contact Help
Advanced Search
Browse by Gene
Browse by Drug
Browse by Pathogen
Enter Keyword:
e.g. Organism, UniProtID, Gene Name......
Search
FOR EXAMPLE :
Pathogen Name: Candida albicans, Aspergillus fumigatus etc.
Host Name: Human, Animal, Plant etc.
Gene Name: ERG11, CYP51a, FKS1 etc.
Gene ID: 3641571, 3509526 etc.
UniProt ID: P10613, Q4WNT5 etc.
Complete AFRbase Data
View all data
Submit
*To see the mutation count categorized by : Gene, Drug and Pathogen Kindly visit 'Statistics' tab in the menu bar .
© Department of Biophysics University of Delhi South Campus.

AFRbase

Antifungal Resistance Database

Home Search Blast 2D View 3D View Submit Statistics Contact Help

Browsing Categories

Browse by Gene

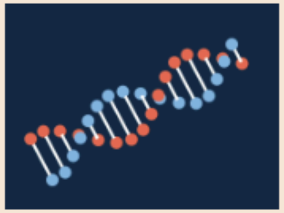

Browse by Drug

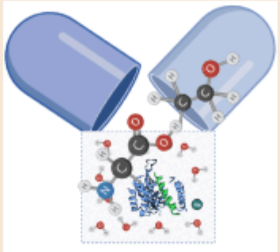

Browse by Pathogen

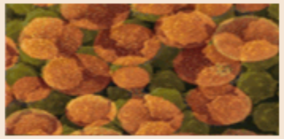

ADVANCED SEARCH

Check the box to Search mutations sorted by Gene

☐CYP51	☐CYP51a
☐CYP51b	☐CYP51c
☐HapE	☐cox10
☐BcSdhB	☐SdhD
☐beta tubulin	☐cytb
☐ERG11	☐FKS1
☐FKS2	☐CYP51
☐MgCYP51	☐DHPS
☐DHFR	☐FUR1
☐GSC1	☐RTA2
☐SQLE	☐Yap1
☐Tac1b	☐PDR1

Submit

AFRbase in Numbers

Host Organisms
3

Fungal species
32

Genes
27

Drugs
37

Diseases
31

Mutations
3688

Research Articles
>6786

Acknowledgements

International Mycological Association, Pfam, NCBI, FRAC, UniProt, gaffi

To cite our database kindly see: Link

© Department of Biophysics University of Delhi South Campus.

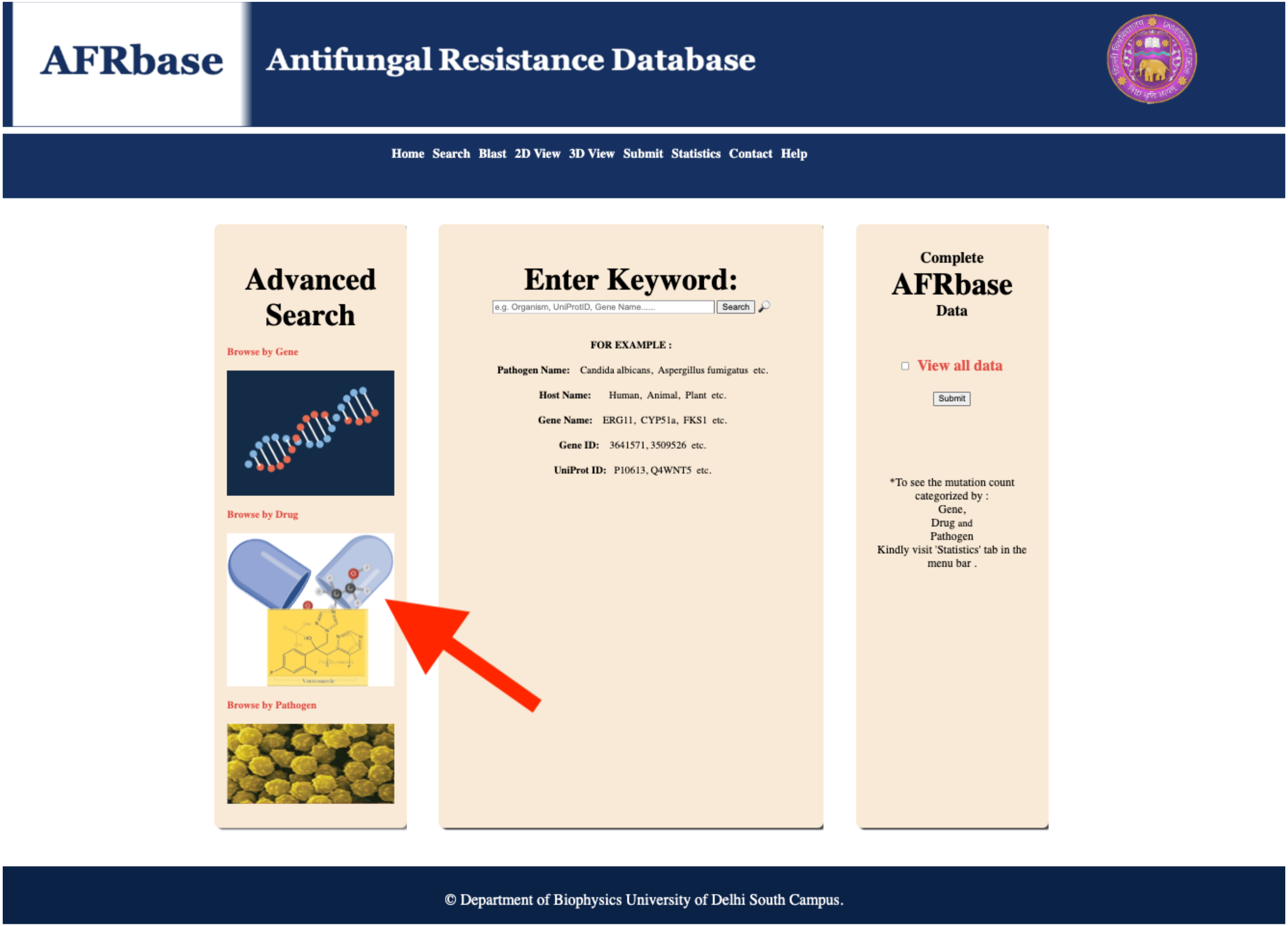
AFRbase
Antifungal Resistance Database
Home Search Blast 2D View 3D View Submit Statistics Contact Help
Advanced Search
Browse by Gene
Browse by Drug
Browse by Pathogen
Enter Keyword:
e.g. Organism, UniProtID, Gene Name......
Search
FOR EXAMPLE :
Pathogen Name: Candida albicans, Aspergillus fumigatus etc.
Host Name: Human, Animal, Plant etc.
Gene Name: ERG11, CYP51a, FKS1 etc.
Gene ID: 3641571, 3509526 etc.
UniProt ID: P10613, Q4WNT5 etc.
Complete AFRbase Data
View all data
Submit
*To see the mutation count categorized by : Gene, Drug and Pathogen Kindly visit 'Statistics' tab in the menu bar .
© Department of Biophysics University of Delhi South Campus.

AFRbase

Antifungal Resistance Database

Home Search Blast 2D View 3D View Submit Statistics Contact Help

Browsing Categories

Browse by Gene

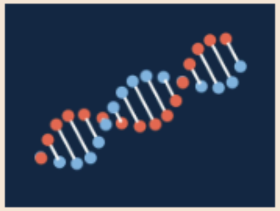

Browse by Drug

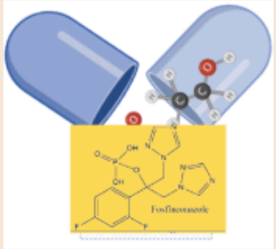

Browse by Pathogen

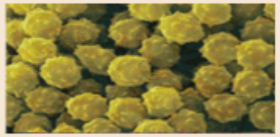

ADVANCED SEARCH

Check the box to Search mutations sorted by Drug

5-flucytosine	Anidulafungin
Arborcandin C	Azoles(FLC VRC POS ITC)
Azoxystrobin	Benzimidazole
Boscalid	Carbendazim
Caspofungin	Cyproconazole
Epoxiconazole	Fenarimol
Fluconazole	Fluqionconazole
Flusilazole	Itraconazole
Metconazole	Micafungin
Pentamidine	Posaconazole
Prochloraz	Propiconazole
Prothioconazole	Pyrimethamine-atovaquone
Pyrimethamine-sulfadoxine	Tebuconazole
Terbinafine	Triflumizole
Trimethoprim-sulfamethoxazole	Voriconazole

Submit

AFRbase in Numbers

Host Organisms
3

Fungal species
32

Genes
27

Drugs
37

Diseases
31

Mutations
3688

Research Articles
>6786

Acknowledgements

International Mycological Association, Pfam, NCBI, FRAC, UniProt, GAFFI

To cite our database kindly see: Link

© Department of Biophysics University of Delhi South Campus.

AFRbase

Antifungal Resistance Database

Home Search Blast 2D View 3D View Submit Statistics Contact Help

Advanced Search

Browse by Gene

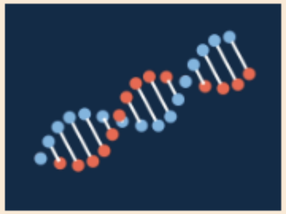

Browse by Drug

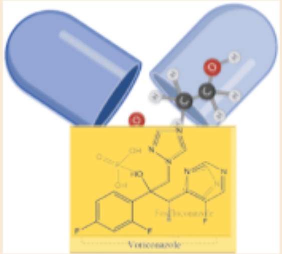

Browse by Pathogen

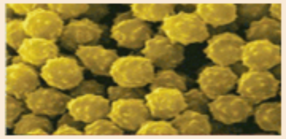

Enter Keyword:

e.g. Organism, UniProtID, Gene Name...... [Search]

FOR EXAMPLE :

Pathogen Name: Candida albicans, Aspergillus fumigatus etc.

Host Name: Human, Animal, Plant etc.

Gene Name: ERG11, CYP51a, FKS1 etc.

Gene ID: 3641571, 3509526 etc.

UniProt ID: P10613, Q4WNT5 etc.

Complete

AFRbase

Data

☐ View all data

[Submit]

*To see the mutation count categorized by :
Gene,
Drug and
Pathogen
Kindly visit 'Statistics' tab in the menu bar .

© Department of Biophysics University of Delhi South Campus.

AFRbase

Antifungal Resistance Database

Home Search Blast 2D View 3D View Submit Statistics Contact Help

Browsing Categories

Browse by Gene

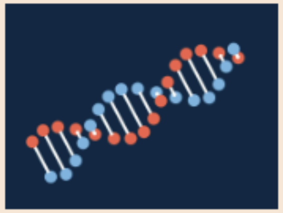

Browse by Drug

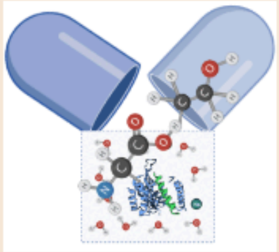

Browse by Pathogen

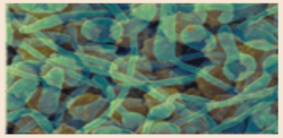

ADVANCED SEARCH

Check the box to Search mutations sorted by Pathogen

☐ Aspergillus flavus	☐ Aspergillus fumigatus
☐ Aspergillus niger	☐ Aspergillus terreus
☐ Botrytis cinerea	☐ Candida albicans
☐ Candida auris	☐ Candida glabrata
☐ Candida krusei	☐ Candida parapsilosis
☐ Candida tropicalis	☐ Cryptococcus neoformans
☐ Erysiphe necator	☐ Fusarium asiaticum
☐ Histoplasma capsulatum	☐ Monilinia fructicola
☐ Mycosphaerella fijiensis	☐ Mycosphaerella graminicola
☐ Oculimacula acuformis	☐ Oculimacula yallundae
☐ Penicillum digitatum	☐ Pneumocytis jirovecii
☐ Puccinia triticina	☐ Pyrenopeziza brassicae
☐ Sclerotinia sclerotiorum	☐ Saccharomyces cerevisiae
☐ Trichophyton interdigitale	☐ Trichophyton mentagrophytes
☐ Trichophyton rubrum	☐ Villosiclava virens
☐ Venturia nashicola	☐ Zymoseptoria tritici

Submit

AFRbase in Numbers

Host Organisms
3

Fungal species
32

Genes
27

Drugs
37

Diseases
31

Mutations
3688

Research Articles
>6786

Acknowledgements

International Mycological Association, Pfam, NCBI, FRAC, UniProt, gaffi

To cite our database kindly see: **Link**

© Department of Biophysics University of Delhi South Campus.

proteininformatics.org/mkumar/afrbase/drug.html

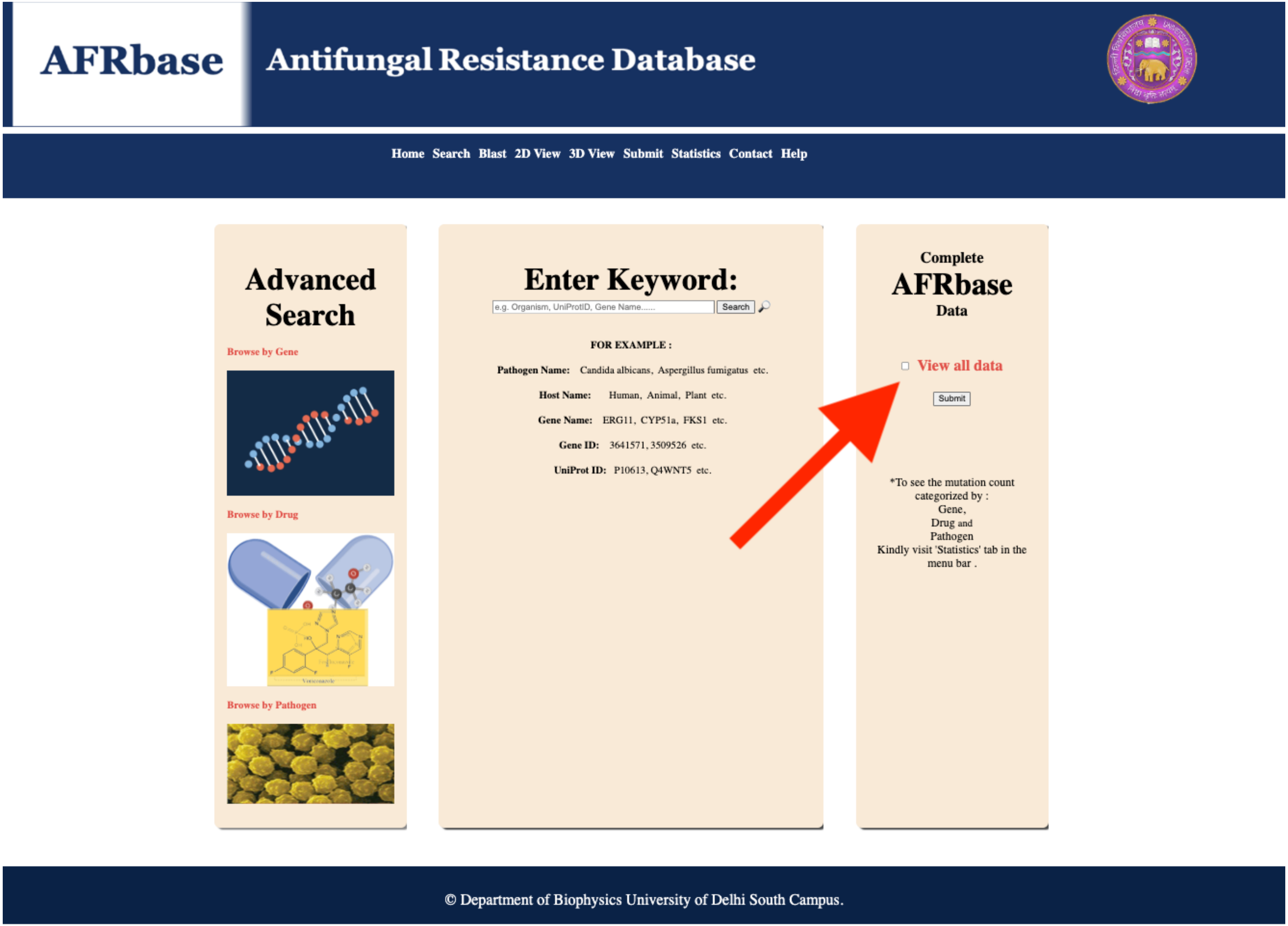
AFRbase
Antifungal Resistance Database
Home Search Blast 2D View 3D View Submit Statistics Contact Help
Advanced Search
Browse by Gene
Browse by Drug
Browse by Pathogen
Enter Keyword:
e.g. Organism, UniProtID, Gene Name......
Search
FOR EXAMPLE :
Pathogen Name: Candida albicans, Aspergillus fumigatus etc.
Host Name: Human, Animal, Plant etc.
Gene Name: ERG11, CYP51a, FKS1 etc.
Gene ID: 3641571, 3509526 etc.
UniProt ID: P10613, Q4WNT5 etc.
Complete AFRbase Data
View all data
Submit
*To see the mutation count categorized by : Gene, Drug and Pathogen Kindly visit 'Statistics' tab in the menu bar .
© Department of Biophysics University of Delhi South Campus.

AFRbase Antifungal Resistance Database

Search Results

Click **AFRbase ID** for more information

Sort results by mutation count per gene

☐ Ascending ☐ Descending

Submit Submit

AFRbase ID	Fungal Pathogen	Host	Gene ID	Gene Name	Mutation Count per gene	Uniprot Entry	AA Mutations	Drug
1	Aspergillus flavus	Human	7912940	CYP51c	2	A0A0H4U5P5	Y319H	Voriconazole
2	Aspergillus flavus	Human	7912940	CYP51c	2	A0A0H4U5P5	T788G	Voriconazole
3	Aspergillus flavus	Human	NA	Yap1	4	NA	L558W	Voriconazole
4	Aspergillus flavus	NA	NA	CYP51	633	NA	G54R	voriconazole
5	Aspergillus flavus	NA	NA	beta-tubulin	172	NA	G54R	voriconazole
6	Aspergillus flavus	NA	NA	ERG11	1357	NA	G54R	voriconazole
7	Aspergillus flavus	NA	NA	CYP51	633	NA	G54R	itraconazole
8	Aspergillus flavus	NA	NA	beta-tubulin	172	NA	G54R	itraconazole
9	Aspergillus flavus	NA	NA	ERG11	1357	NA	G54R	itraconazole
10	Aspergillus flavus	NA	NA	CYP51	633	NA	G54R	posaconazole
11	Aspergillus flavus	NA	NA	beta-tubulin	172	NA	G54R	posaconazole
12	Aspergillus flavus	NA	NA	ERG11	1357	NA	G54R	posaconazole
13	Aspergillus flavus	NA	NA	CYP51	633	NA	G54R	azole
14	Aspergillus flavus	NA	NA	beta-tubulin	172	NA	G54R	azole
15	Aspergillus flavus	NA	NA	ERG11	1357	NA	G54R	azole
16	Aspergillus flavus	NA	NA	CYP51	633	NA	G54R	isavuconazole
17	Aspergillus flavus	NA	NA	beta-tubulin	172	NA	G54R	isavuconazole
18	Aspergillus flavus	NA	NA	ERG11	1357	NA	G54R	isavuconazole
19	Aspergillus flavus	NA	NA	CYP51	633	NA	G54R	fluconazole
20	Aspergillus flavus	NA	NA	beta-tubulin	172	NA	G54R	fluconazole

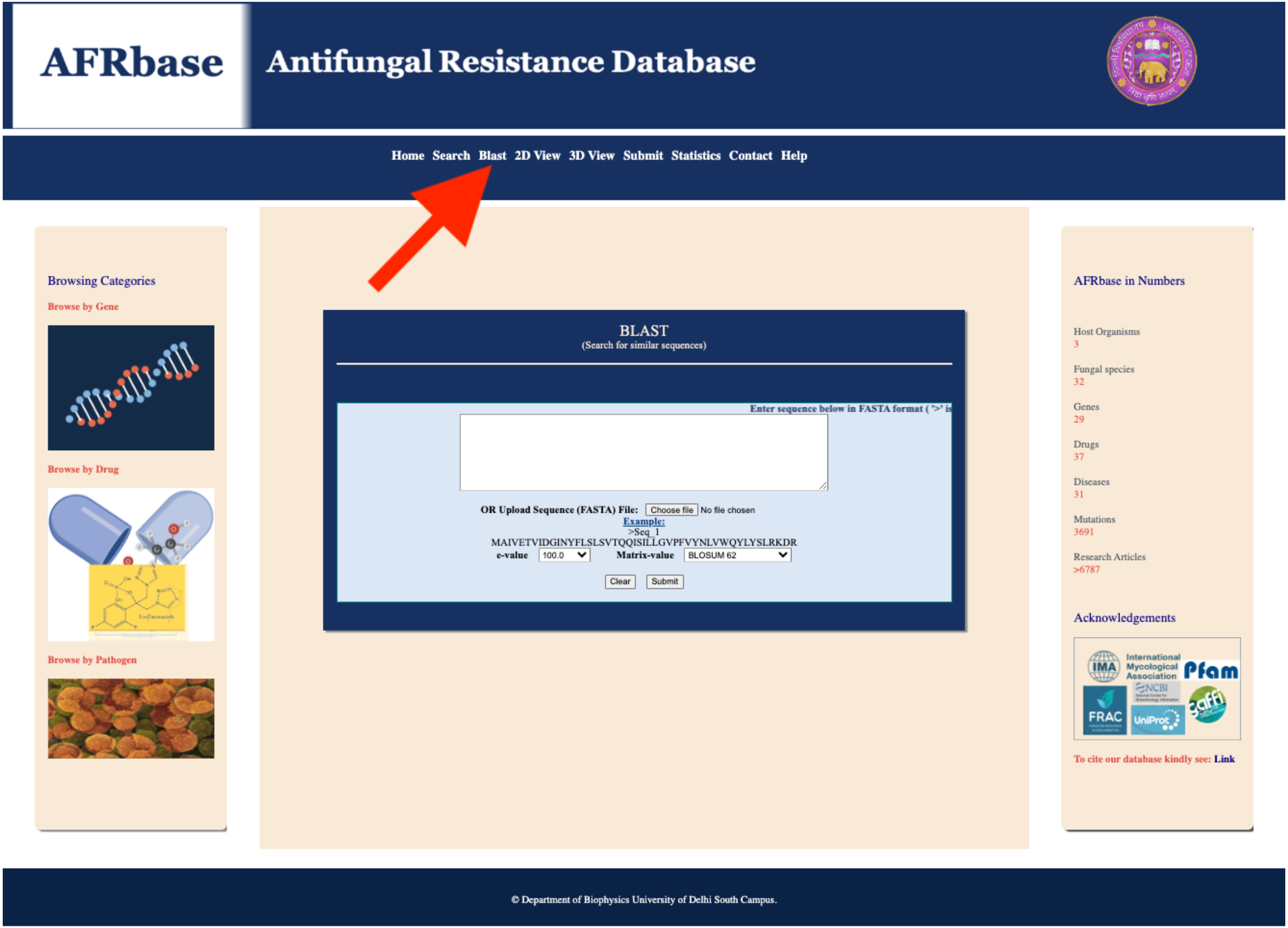
AFRbase
Antifungal Resistance Database
Home Search Blast 2D View 3D View Submit Statistics Contact Help
Browsing Categories
Browse by Gene
Browse by Drug
Browse by Pathogen
BLAST
(Search for similar sequences)
Enter sequence below in FASTA format ('>' is
OR Upload Sequence (FASTA) File: Choose file No file chosen
Example:
>Seq_1
MAIVETVIDGINYFLSLSVTQQISILLGVPFVYNLVWQYLYSLRKDR
e-value 100.0 Matrix-value BLOSUM 62
Clear Submit
AFRbase in Numbers
Host Organisms
3
Fungal species
32
Genes
29
Drugs
37
Diseases
31
Mutations
3691
Research Articles
>6787
Acknowledgements
To cite our database kindly see: Link
© Department of Biophysics University of Delhi South Campus.

BLAST RESULTS

BLASTP 2.2.26
Reference:
Altschul, StephenF., Thomas L. Madden, Alejandro A. Schaffer,Jinghui Zhang, Zheng Zhang, Webb Miller, and David J. Lipman (1997), Gapped BLASTand PSI-BLAST: a new generation of protein database search programs, Nucleic Acids Res. 25:3389-3402.
Reference for compositional score matrix adjustment:
Altschul, Stephen F., John C. Wootton, E. Michael Gertz, Richa Agarwala, Aleksandr Morgulis, Alejandro A. Schaffer, and Yi-Kuo Yu (2005) Protein database searches using compositionally adjusted substitution matrices, FEBS J. 272:5101-5109.

```
BLASTP 2.2.26 [Sep-21-2011]

Query= Seq_1
         (47 letters)
Database: afrbase_sequences_2line.fasta
           22 sequences; 17,999 total letters

Searching.................................................done
                                                                 Score    E
Sequences producing significant alignments:                      (bits) Value
P10613                                                              97   1e-28
Q58J66                                                              80   9e-23
A0A411K953                                                          56   3e-14
Q6FTN8                                                              17   1.9
Q6FMZ3                                                              17   2.0
A0A1D8PCT0                                                          17   3.5
Q18ND9                                                              15   8.3
Q4U2W2                                                              15   9.2
Q4WP81                                                              15      11
Q4WLT4                                                              15      15
Q4WNT5                                                              14      37
A0A0H4U5P5                                                          13      59
```

P10613

```
          Length = 528
 Score = 96.7 bits (239), Expect = 1e-28,   Method: Compositional matrix adjust.
 Identities = 47/47 (100%), Positives = 47/47 (100%)
Query: 1  MAIVETVIDGINYFLSLSVTQQISILLGVPFVYNLVWQYLYSLRKDR 47
          MAIVETVIDGINYFLSLSVTQQISILLGVPFVYNLVWQYLYSLRKDR
Sbjct: 1  MAIVETVIDGINYFLSLSVTQQISILLGVPFVYNLVWQYLYSLRKDR 47
 Score = 18.1 bits (35), Expect = 0.88,   Method: Compositional matrix adjust.
 Identities = 6/7 (85%), Positives = 6/7 (85%)
Query: 31  FVYNLVW 37
           FVYNL W
Sbjct: 487 FVYNLRW 493
 Score = 12.7 bits (21), Expect =    98,   Method: Compositional matrix adjust.
 Identities = 4/6 (66%), Positives = 5/6 (83%)
Query: 23  ISILLG 28
           I IL+G
Sbjct: 302 IGILMG 307
```

Q58J66

```
          Length = 528
 Score = 80.1 bits (196), Expect = 9e-23,   Method: Composition-based stats.
 Identities = 36/47 (76%), Positives = 42/47 (89%)
Query: 1  MAIVETVIDGINYFLSLSVTQQISILLGVPFVYNLVWQYLYSLRKDR 47
          MAIV+T IDGINYFLSLS+TQQI+IL+  PF+YN+ WQ LYSLRKDR
```

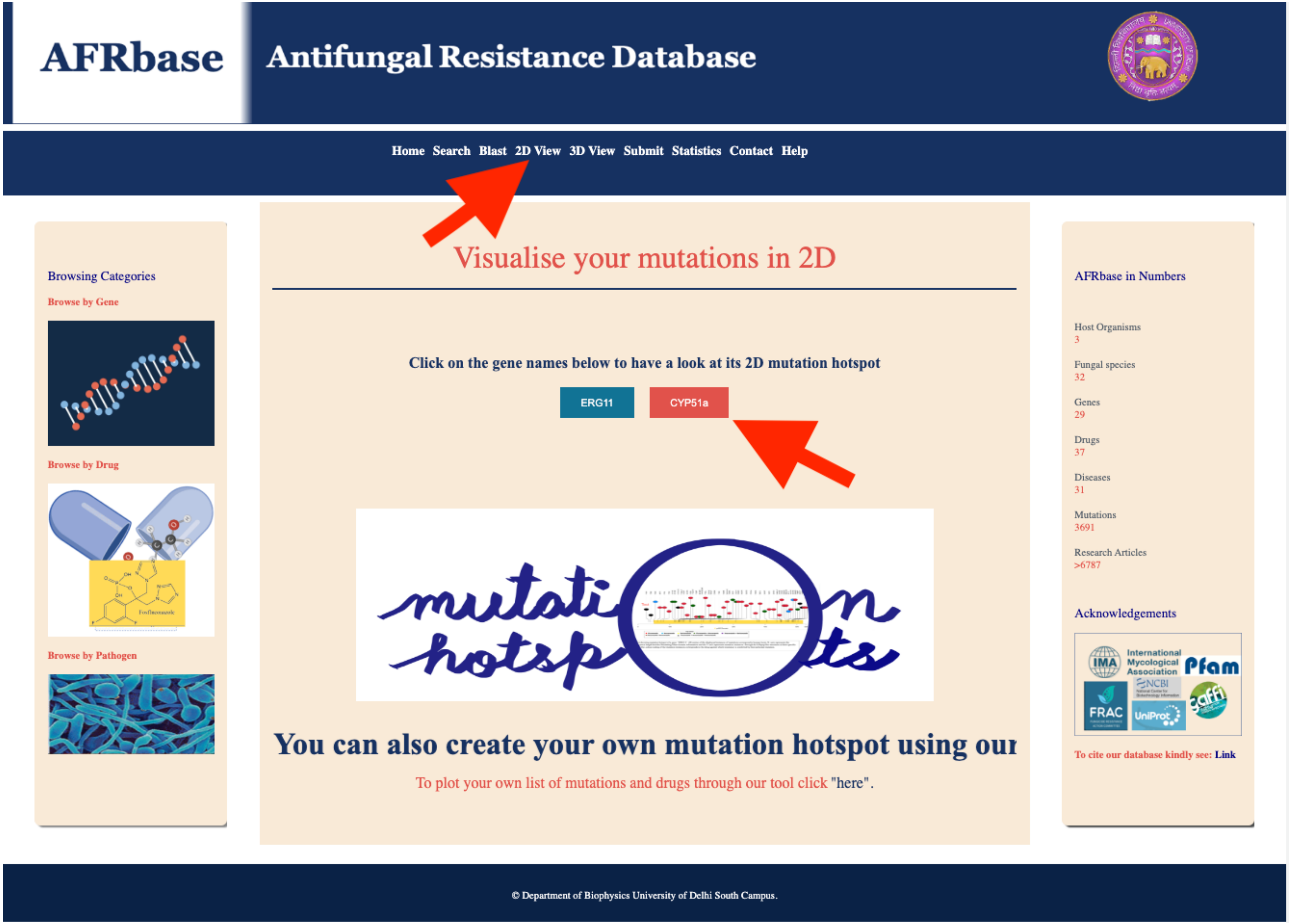
AFRbase
Antifungal Resistance Database
Home Search Blast 2D View 3D View Submit Statistics Contact Help
Browsing Categories
Browse by Gene
Browse by Drug
Browse by Pathogen
Visualise your mutations in 2D
Click on the gene names below to have a look at its 2D mutation hotspot
ERG11
CYP51a
mutation hotspots
You can also create your own mutation hotspot using our
To plot your own list of mutations and drugs through our tool click "here".
AFRbase in Numbers
Host Organisms
3
Fungal species
32
Genes
29
Drugs
37
Diseases
31
Mutations
3691
Research Articles
>6787
Acknowledgements
IMA International Mycological Association
Pfam
NCBI
FRAC
UniProt
To cite our database kindly see: Link
© Department of Biophysics University of Delhi South Campus.

AFRbase

Antifungal Resistance Database

Home Search Blast 2D View 3D View Submit Statistics Contact Help

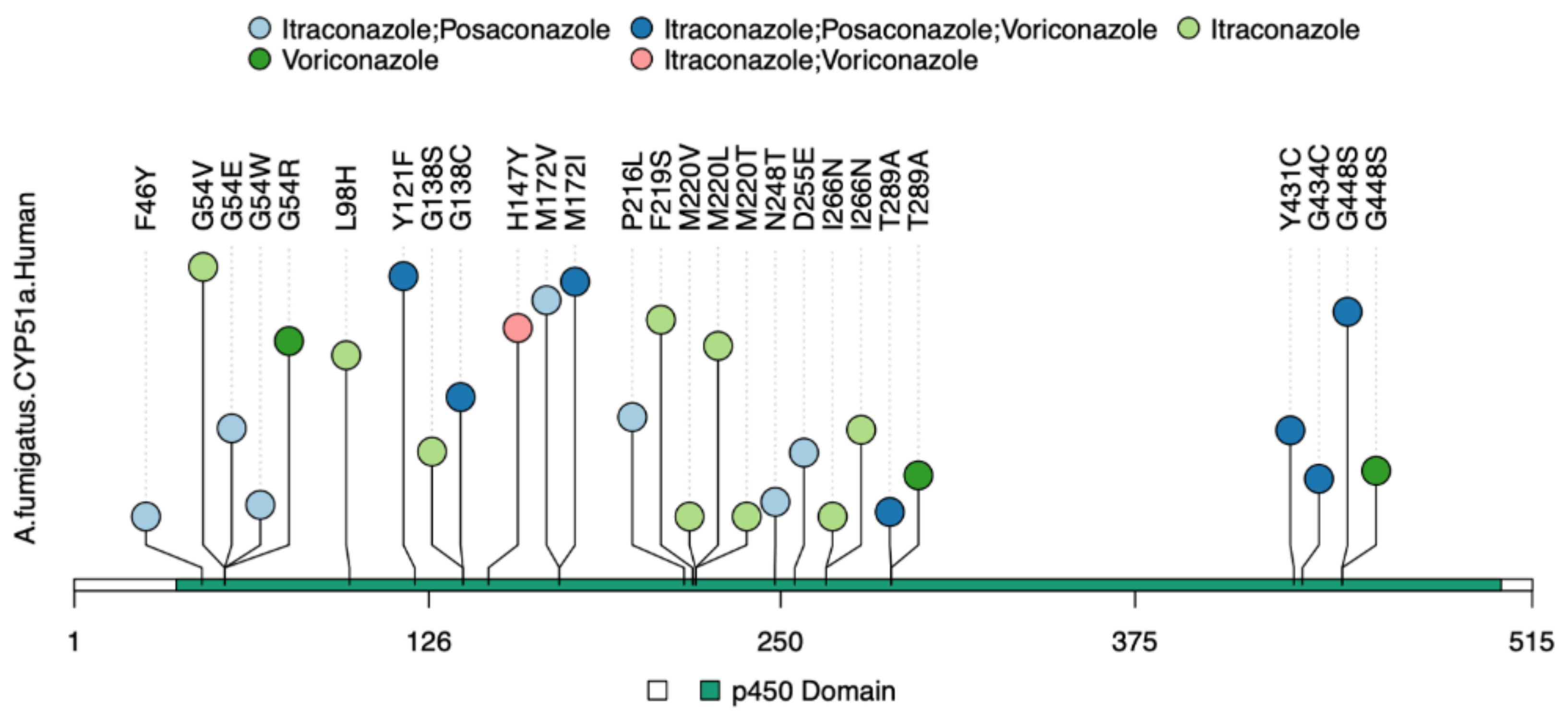

Plot showing mutation hotspot of a gene- 'CYP51a' of *Aspergillus fumigatus*. All entries of the displayed instances of mutations correspond to human hosts. X-axis represents the sequence length besides illustrating Pfam domain information and the Y-axis represents mutation instances through the lollipop like structures at their specific loci. The colour coding of the mutation instances corresponds to the drug against which resistance is conferred by that particular mutation.

© Department of Biophysics University of Delhi South Campus.

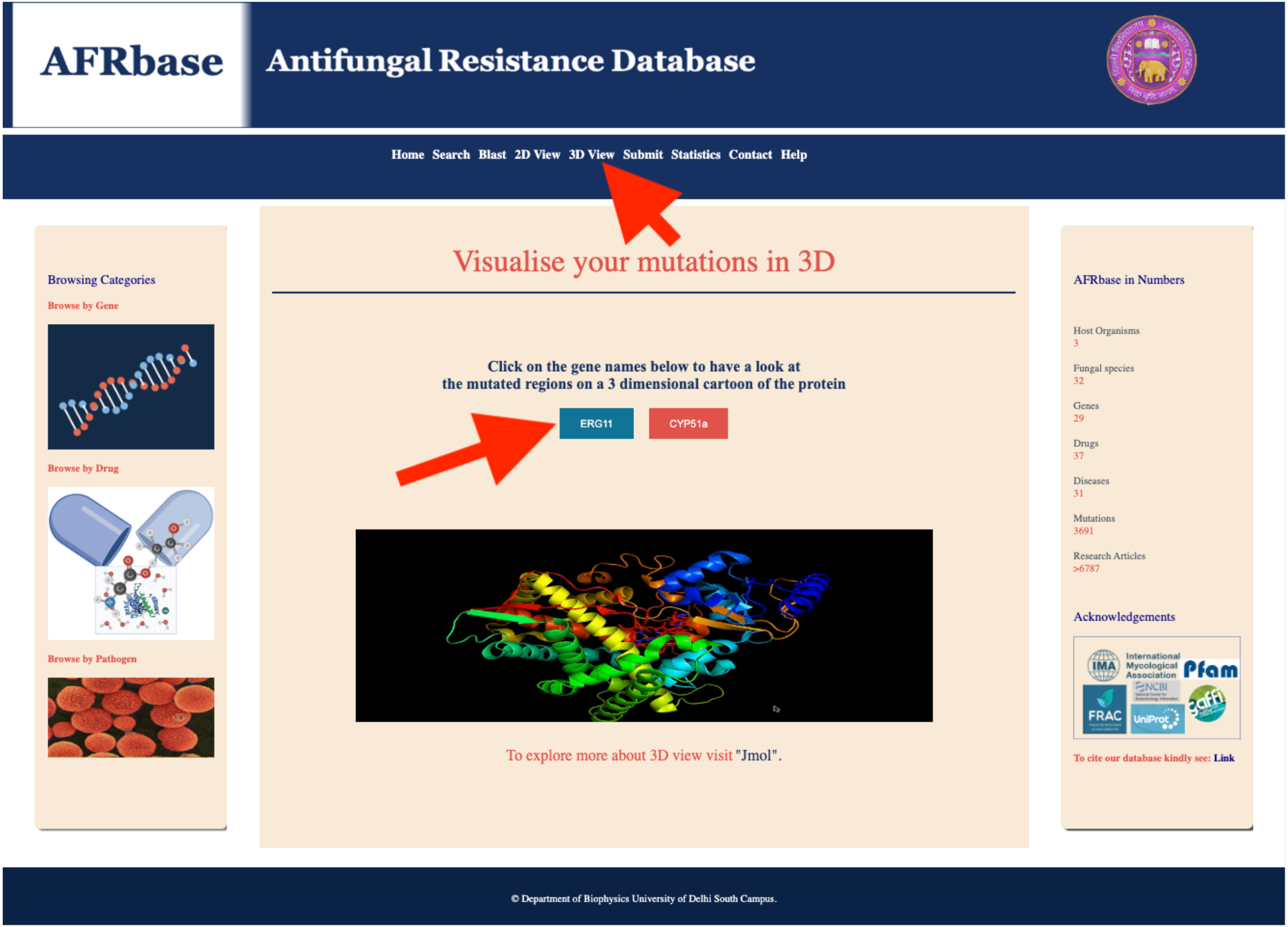
AFRbase
Antifungal Resistance Database
Home Search Blast 2D View 3D View Submit Statistics Contact Help
Browsing Categories
Browse by Gene
Browse by Drug
Browse by Pathogen
Visualise your mutations in 3D
Click on the gene names below to have a look at
the mutated regions on a 3 dimensional cartoon of the protein
ERG11
CYP51a
To explore more about 3D view visit "Jmol".
AFRbase in Numbers
Host Organisms
3
Fungal species
32
Genes
29
Drugs
37
Diseases
31
Mutations
3691
Research Articles
>6787
Acknowledgements
International Mycological Association
IMA
Pfam
NCBI
FRAC
UniProt
To cite our database kindly see: Link
© Department of Biophysics University of Delhi South Campus.

AFRbase

Antifungal Resistance Database

View mutations in your protein of interest in 3D

Gene Name: ERG11
PDB ID: 5v5z
Uniprot ID: P10613
Pathogen: Candida albicans
The mutations reported in this protein has been colour coded according to the drug catalogue specified below. For example : the red coloured region in the protein are mutations which confer resistance against the drug Fluconazole. (Note: The catalogue is common for 2D and 3D visualisation)

Drug catalogue

- Fluconazole.
- Itraconazole
- Voriconazole.
- Fluconazole + Itraconazole
- Itraconazole + Voriconazole
- Fluconazole+ Voriconazole
- Fluconazole + Itraconazole + Voriconazole

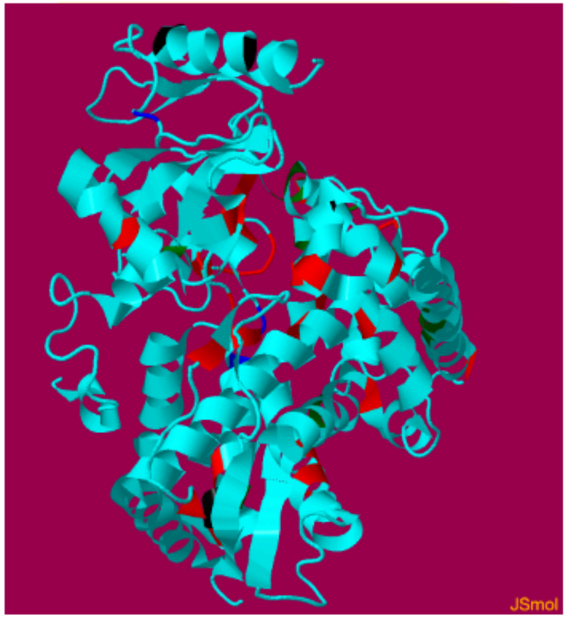

Spin on
background color:
Stereo Off
Animation
Mode:
Jmol Console

CLICK IMAGE TO ACTIVATE 3D
The colour key of the mutations is given alongside.

© Department of Biophysics University of Delhi South Campus.

AFRbase

Antifungal Resistance Database

Home Search Blast 2D View 3D View Submit Statistics Contact Help

Browsing Categories

Browse by Gene

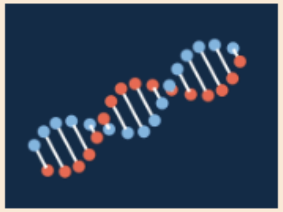

Browse by Drug

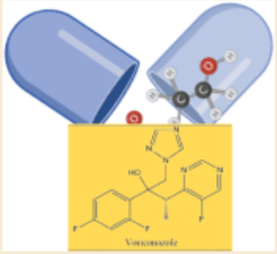

Browse by Pathogen

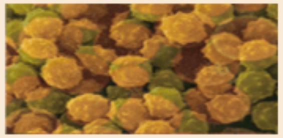

Statistics

Gene	No.of mutations recorded
ERG11	1357
CYP51	633
fks1	319
FKS2	206
beta-tubulin	172
CYP51a	57
HapE	51
GSC1	42
MgCYP51	12
CYP51b	10
DHFR	8
MfCYP51	7
cox10	5
Yap1	4
BcSdhB	3
DHPS	3
SQLE	3
CYP51c	2
FUR1	2
tub2	2
NA	787
OTHERS	6
**Grand Total**	**3691**

Drug	No.of mutations recorded
azole	858
fluconazole	645
itraconazole	429
Voriconazole	390
caspofungin	275
posaconazole	211
anidulafungin	208
micafungin	208
isavuconazole	71
ketoconazole	70
flucytosine	65
terbinafine	44
miconazole	27
Prochloraz	23
clotrimazole	22
miltefosine	16
rezafungin	15
Propiconazole	9
luliconazole	8
olorofim	8
Triflumizole	8
Epoxiconazole	6
Boscalid	5
econazole	5
Pyrimethamine-sulfadoxine	5
OTHERS	60
**Grand Total**	**3691**

Fungal Pathogen	No.of mutations recorded
Candida albicans	988
Aspergillus fumigatus	624
Candida glabrata	534
Candida tropicalis	351
Candida parapsilosis	196
Candida auris	188
Candida krusei	175
Aspergillus flavus	132
Cryptococcus neoformans	88
Aspergillus terreus	40
Aspergillus niger	17
Penicillum digitatum	14
Mycosphaerella graminicola	12
Pneumocytis jirovecii	11
Pyrenopeziza brassicae	10
Mycosphaerella fijiensis	7
Oculimacula yallundae	7
Oculimacula acuformis	6
Saccharomyces cerevisiae	5
Botrytis cinerea	4
Zymoseptoria tritici	3
NA	265
OTHERS	14
**Grand Total**	**3691**

Expand others

AFRbase in Numbers

Host Organisms
3

Fungal species
32

Genes
29

Drugs
37

Diseases
31

Mutations
3691

Research Articles
>6787

Acknowledgements

To cite our database kindly see: Link

© Department of Biophysics University of Delhi South Campus.
